# Study of the Viscoelastic and Rheological Properties of Rubber-Bitumen Binders Obtained from Rubber Waste

**DOI:** 10.3390/polym16010114

**Published:** 2023-12-29

**Authors:** Anar Akkenzheyeva, Viktors Haritonovs, Akkenzhe Bussurmanova, Remo Merijs-Meri, Yerzhan Imanbayev, Arturs Riekstins, Akmaral Serikbayeva, Serik Sydykov, Murshida Aimova, Gulnara Mustapayeva

**Affiliations:** 1 Engineering Faculty, Yessenov University, 32 Microdistrict, Aktau 130003, Kazakhstan; anar.akkenzheyeva@yu.edu.kz (A.A.); akmaral.serikbayeva@yu.edu.kz (A.S.); 2 Faculty of Civil and Mechanical Engineering, Riga Technical University, 6A Kipsalas Street, LV-1048 Riga, Latvia; viktors.haritonovs@rtu.lv (V.H.); arturs.riekstins_2@rtu.lv (A.R.); 3 Science and Technology Faculty, Yessenov University, 32 Microdistrict, Aktau 130003, Kazakhstan; murshida.aimova@yu.edu.kz (M.A.); gulnara.mustapayeva@yu.edu.kz (G.M.); 4 Faculty of Natural Sciences and Technologies, Riga Technical University, 3 Paula Valdena Street, LV-1048 Riga, Latvia; remo.merijs-meri@rtu.lv; 5 Laboratory of Petrochemical Processes, RSE «Institute of Combustion Problems», Bogenbay Street, 172, Almaty 050012, Kazakhstan; erzhan.imanbayev@mail.ru; 6 LLP “JV“ CASPI BITUM Aktau Bitumen Plant, Industrial Zone 5, Building 65, Aktau 130000, Kazakhstan; s.sydykov82@mail.ru

**Keywords:** petroleum bitumen, modification, physical and mechanical characteristics, crumb rubber, rheological properties

## Abstract

According to scientific research from different countries, crumb rubber obtained from end-of-life tires (ELT) during processing can improve the properties of the asphalt mixture, thereby extending the service life of the road surface. This paper presents the modification of bitumen with industrial rubber waste. The modification of bitumen for roads is considered one of the most suitable and popular approaches. This research paper describes the details of using different types of crumb rubber as bitumen modifiers. The modified bitumen’s main physical and mechanical characteristics were determined after conventional tests: penetration and ductility, softening point, and Fraas brittleness point. In order to obtain a rubber–asphalt concrete mixture with improved performance characteristics, the viscoelastic and rheological properties of rubber–bitumen binders and their comparison with polymer–bitumen binders were also studied. The research results show that with increasing temperature, the values of viscosity, shear stress and complex shear modulus of all studied bitumen systems decrease, the values of the phase shift angle increase, and the size of the rubber particles has a greater influence on the properties of bitumen systems.

## 1. Introduction

Rubber waste is generated in large quantities while producing consumer goods and in the tire industry. In 2019, 324 million tires were sold in Europe alone [1,2]. Currently, it can be assumed that approximately the same number of tires become used tires per year. In addition, a significant number of these tires are already in warehouses and landfills, although this varies from country to country [3,4,5,6,7,8,9,10,11,12].

Waste tires in landfills and warehouses pose a serious environmental problem due to their high toxicity and flammability since tires are flammable. Uncontrolled tire fires can produce a large amount of smoke and toxic air pollutants, including benzene and polycyclic aromatic hydrocarbons [13]. Tires in landfills, dumps, or warehouses can take a very long time to decompose, releasing the toxic metals cadmium, lead and zinc. In addition, there is a possibility of toxic chemicals leaching into ground or surface water [14]. Unlike some other types of waste, such as wood waste, plant waste, and food industry waste, rubber waste is practically not biodegradable, so it must be disposed of. Currently, the method of recycling waste tires is combustion [15,16]. This process results in the release of chemicals such as chlorinated methane, dioxins, and polychlorinated biphenyls. In addition, with strong heating, pyrolytic oil is formed, polluting the soil, surface, and groundwater. It is worth noting that emissions from burning waste tires are much more mutagenic than those from coal-fired power plants [17]. Thus, the disposal of these tires poses a major environmental and economic problem in almost all developed countries [18,19].

The viewpoint that ELTs are just solid waste has changed, and they are now considered a material that can be widely reused in various ways and applications [20]. To solve the problem, a significant amount of ELT is used for energy production, and regeneration (for example, in cement factories), but the potential of the valuable material is not fully used. In 2017, up to 35% of ELT was used for energy production in Europe [21,22]. A more promising way to use ELT is to obtain crushed crumb rubber from them (CR—Crumb Rubber), which can be used for various purposes, for example, as a modifier (CRM—Crumb Rubber Modifier) to improve the properties of bitumen and asphalt concrete mixtures. Research on using crumb rubber from waste tires on road surfaces has been found since the 1960s. Regulating the operational properties of bitumen binders by introducing into them a modifier based on crumb rubber from the processing of worn-out tires is currently the most promising and successfully developing area of bitumen modification. According to scientific research in different countries, crumb rubber obtained from ELT during processing can improve the properties of the asphalt mixture, thereby extending the service life of the road surface [23,24,25,26,27,28].

The two most popular methods of introducing crumb rubber into an asphalt mixture are the wet method and the dry method. The wet process is the modification of bitumen with rubber, while the dry process is a method in which rubber particles are directly added to the asphalt mixture with the main purpose of replacing small amounts of aggregate, such as sand particles. Today, the goal of both methods is to produce asphalt concrete with improved characteristics. However, the wet process seems more promising since the interaction of bitumen and rubber is more direct and intense in it [28,29,30]. However, some researchers claim that it is possible to obtain similar performance characteristics from both methods [31,32,33].

Both methods have their advantages and disadvantages. The wet process requires expensive equipment to modify the bitumen and poor storage stability has been observed in some cases. With the wet method, the content of CR crumb rubber can reach up to 25% of the bitumen mass [33,34], and the recommended particle size is up to 2 mm [31]. The dry process is easier to implement since no special equipment is required (only adaptation of the asphalt plant may be required) since the crumb rubber is added directly to the mixer of the asphalt plant [31]. Unlike the wet process, the reaction time between the bitumen and the rubber particles is shorter and less intense, so the dry process requires smaller rubber particles (less than 0.8 mm) to achieve the same effect. It takes more energy to produce such a small crumb rubber. The wet process produces an asphalt binder with too high a viscosity, but the dry process has too short of a mixing time for interaction between the rubber particles and the bitumen to occur [35]. Unfortunately, higher mixing temperatures result in increased amounts of vapors and pollutants, such as emissions of xylene, toluene, and other volatile organic compounds [36].

Currently, there are two different wet process technologies: high viscosity wet process and non-stirring wet process [31]. The high-viscosity wet process completely incorporated the crumb rubber into the asphalt binder itself. This makes the process suitable for the production of both modified asphalt binders and spray binders [22]. In this process, asphalt rubber is produced by the reaction between finely ground tire rubber and traditional asphalt binders. This process improves the adhesive properties of binders when added to fine and coarse aggregates in the production of road asphalt pavements [37]. Pre-mixing crumb rubber and asphalt binder can be done at an asphalt plant or on-site using portable mixing plants [38].

Although ground tire rubber reacts with an asphalt binder before being mixed with aggregates in a wet process, it has been shown that the viscosity of the final product begins to decrease after approximately 2 h [39]. The decrease in viscosity is believed to be due to incomplete degradation of the crumb rubber in the asphalt binder and compatibility problems. As a result, modified asphalt binders produced using a high-viscosity wet process must be constantly mixed during transportation and storage to avoid phase separation and maintain the desired properties [38].

To overcome the storage problems associated with the high-viscosity wet process, the production method can be modified to produce a product that will not separate over time. The terminal blend technology is an improved version of the high-viscosity wet process that uses higher pressures, mixing temperatures, and shear forces to ensure the complete breakdown of shredded tire rubber particles into asphalt binder [40]. In this process, complete decomposition is achieved through decomposing and devulcanizing crumb rubber particles under mixing conditions. The wet, terminal blend technology process produces a modified binder that is more uniform and noticeably smoother than the high-viscosity process. In addition, improved breakdown of crumb rubber particles results in better binder storage properties, and mixing during transportation and storage is not required [40].

In the wet process, CR tire rubber and bitumen react at high temperatures (usually 18 to 26% rubber is added relative to the weight of bitumen). This process, when CR disperses and swells in the mixture, is greatly influenced by the mixing temperature, mixing time, CR size, morphology, the amount of aromatic compounds present in the bitumen, and the type and energy of mechanical mixing performed.

The rate of reaction between the crumb rubber and bitumen is mainly affected by temperature and mixing time [41] and can, therefore, be optimized by controlling these, as well as other process variables listed previously [42,43,44,45]. To obtain a rubber–asphalt concrete mixture with improved performance characteristics, rotational viscosity is the physical parameter that is easiest to control during the modification process from an operational point of view. In fact, the interaction of rubber with bitumen at a certain temperature implies an increase in the viscosity of the mixture to a maximum value and a subsequent noticeable decrease with reaction time [46]. It should also be noted that the addition of a crumb rubber can initiate the non-Newtonian behavior of the bitumen binder and impart a yield strength due to the fact that systems filled with devulcanized crumb rubber have a structure formed by filler particles in a bulk material [35]. Thus, the dry process is easier to carry out, while the wet process, although more complex, has the advantage of allowing precise control of the rheological properties of the resulting binder.

The results of numerous studies have shown that the running characteristics of bitumen-based compositions are closely related to their rheological parameters [47,48,49,50,51,52].

It was indicated in [53] that the specificity of the inter-component interaction affects the viscoplastic and relaxation properties of bitumen binders and determines the stability of the material. Many modifying additives are incompatible or only kinetically compatible with the liquid components of bitumen, which leads to phase separation after prolonged exposure to the operating temperatures of the material. In addition, the authors of [35] showed that the addition of relatively small amounts of devulcanized crumb rubbers to bitumen binders improves their viscoelastic properties and rubber–bitumen binders are characterized by a monotonous decrease in viscoelastic properties with increasing temperature.

Rheological properties, depending on the temperature of the experiment, characterize modified bitumen: (1) during its production; (2) when mixed with minerals and other additives of particles of different sizes when producing asphalt concrete; (3) when laying the pavement; and (4) by performance over the entire service life of the pavement. Assessing the rheological properties of a rubber asphalt binder is fundamental to assessing the design life of a pavement and its extension when crumb rubber is used to lay flexible pavements [31].

In search of a more profitable economic and environmental solution, the purpose of the research work is to study the viscoelastic and rheological properties of rubber–bitumen binders obtained using non-vulcanized crumb rubber with larger size particles, the production of which costs EUD 150/ton, while traditional rubber powder is up to EUD 600–800 per ton. A comparison was made with the rheological properties of rubber–bitumen binders obtained using non-vulcanized crumb rubber of different sizes and origins, as well as the rheological properties of polymer–bitumen binders.

## 2. Materials and Methods

This study used B70/100-grade bitumen to prepare modified bitumen. Characteristics of petroleum road bitumen grade B70/100 are given in Table 1.

Crumb rubber obtained from used car tires (SR100-truck tires, VR100-passenger car tires, SR50/VR50-the equivalent mix of truck and passenger car tires) were used: (a) coarse fraction (2.5–3.5 mm) and (b) fine fraction (2.0–2.5 mm). Additionally, extra-fine fraction (0.8 mm) was used. All fractions of crumb rubber are from the Eco Baltia vide company.

Commercial styrene–butadiene–styrene elastomer (SBS-01-10) was also used for bitumen modification. The properties of the SBS polymer are given in Table 2.

**Figure 1:**
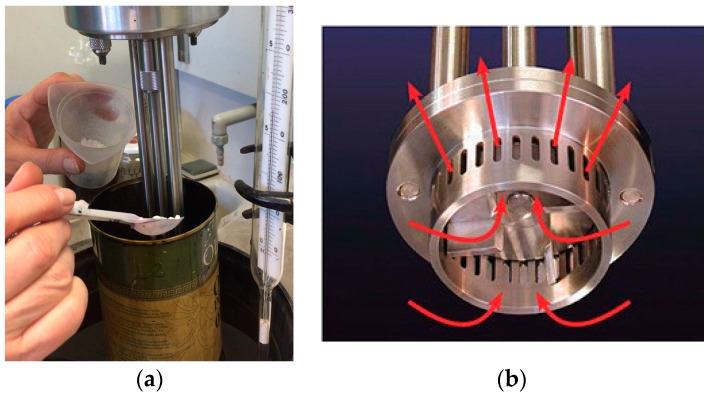
Bitumen modified with crumb rubber: (**a**) the acquisition process; (**b**) circulation of the flow during the mixing process (red arrows denote to the bituminous mass circulation pattern during mixing).

The process of obtaining modified bitumen is presented in Figure 1. In this study, the development of different modified bitumen (MB) formulations was carried out in laboratory conditions using the “wet” method.

The modified bitumen was prepared as follows: initially, the bitumen was heated to 140 ± 1 °C in a laboratory oven, then the vessel with the flowable bitumen was placed in a bath of silicone oil and, when the temperature reached 170 ± 1 °C, the modifier particles were gradually added and stirred for a specified time at a temperature of 190 ± 1 °C and speeds of 6000–7000 rpm using a high shear rotary laboratory mixer equipped with a square mesh screen, model Silverson L5M-A (UK).

The physical properties of B70/100 base bitumen and the modified bitumen formulations were tested using conventional industry tests such as needle penetration (EN 1426 [54]), softening point (EN 1427 [55]), elastic recovery (EN 13398 [60]), Fraass (EN 12593 [56]).

Characterization of viscoelastic properties was carried out in accordance with international industry standards, including EN 14770 [61], AASHTO TP 70 [62] and ASTM D7405 [63]. Rheological properties were determined using Anthon Paar SmartPave 102 dynamic shear rheometer (DSR) with a 25-plate configuration at 10 rad/s oscillation and 12% shear strain. The test procedure, according to EN 14770 [61], involves determining the viscoelastic properties of bitumen (including complex shear modulus G* and phase shift angle δ) at different temperatures, which are gradually increased in steps of 6 °C, to determine the critical temperature at which the bitumen reaches a certain limit value (for example, 1 kPa for an unaged bitumen system, estimated by the parameter |G*|/sinδ). Elastic recovery was determined using multiple stress creep recovery (MSCR) tests according to AASHTO TP 70 [62] and ASTM D7405 [63]. The test involves cyclic loading—unloading of the bitumen sample at two different successive stress levels (10 cycles at 0.1 kPa followed by 10 cycles at 3.2 kPa) at the selected temperature.

## 3. Results and Discussion

In the process of modifying a bitumen binder with a crumb rubber, it was discovered that they swell significantly during modification (because of the saturation of the crumb rubber with maltenes, the main component of bitumen). This significantly impairs the ease of processing (workability) of the bitumen—the ability of the modified bitumen to mix well with crushed stone, and also makes it difficult to pump and transfer during production due to its high viscosity. This effect was especially pronounced when large fractions (2.5–3.5 mm) of rubber of various origins were introduced into bitumen.

The particle size of crumb rubber affects the rheological properties of bitumen modified with crumb rubber [63]. Smaller particle sizes produce modified bitumen that has higher viscosity, softening point and elasticity due to its larger surface area [64,65,66,67] and aspect ratio [68]. Higher viscosity also results in better rutting resistance, as shown by the authors [68]. On the other hand, the authors [69] showed that larger crumb rubber particle size provided better performance at high temperatures and similar cracking resistance at low temperatures compared to the CRMB manufactured with smaller crumb rubber particle size.

Therefore, in the process of modifying the bitumen binder, crumb rubber with a size of 2.0–2.5 mm was selected. On the other hand, in order to find out how reducing the particle size of crumb rubber affects the properties of bitumen, bitumen compositions modified with crumb rubber with even smaller sizes were simultaneously studied. The size of the rubber particles was 0.8 mm.

In order to determine the effect of the amount of crumb rubber on the rheological properties and ease of processing (workability) of the bitumen binder, compositions with a concentration of crumb rubber from 15 to 25% were created. In general, from a recycling perspective, it is attractive to incorporate as much waste as possible into the bitumen, resulting in a higher recovery rate. However, taking into account that with an increase in the concentration of rubber particles, the ease of operation of the system decreases, the predicted energy costs and, consequently, the preliminary costs of the technological process increase, more detailed studies of the properties were carried out for compositions with the lowest concentration of crumb rubber in bitumen—15%. It was also noted in [35] that the addition of crumb rubber causes an exponential increase in the viscosity of bitumen, which limits the possible crumb content in bitumen for technological reasons.

The most suitable technology for the production of bitumen modified with crumb rubber in laboratory conditions has been developed by varying the pre-treatment conditions of crumb rubber—pre-swelling, mixing speed profile (3000–8000 rpm), processing temperature (180–200 °C), and processing time (0.5–5 h). It has been established that the acceptable technological regime under laboratory conditions is as follows: (1) mixing temperature—~200 °C; (2) range of mixing speeds with a high-intensity mixer—6000–7000 rpm; and (3) processing time—1–2 h (without swelling).

“Road Conditions 2019” allows the use of bitumen modified with crumb rubber from outworn tires, provided that the properties of such a binder are similar to those of polymer-modified bitumen. Manufacturers usually prefer to use bitumen modified with SBS polymers.

The performance characteristics of bitumen systems modified with crumb rubber were compared with unmodified bitumen, as well as with the performance characteristics of a bitumen system modified with 4% commercial styrene-butadiene-styrene thermoplastic elastomer (SBS) additive. The concentration of the SBS additive corresponds to the currently used traditional recipes of polymer-modified bitumen (PMB) to obtain PMB type 45/80-55 in accordance with the requirements of the EN 14023 [64] standard and the “Road Specifications 2019”.

Table 3 shows the most important properties of the original bitumen, bitumen modified with SBS, and bitumen modified with rubber additives.

The results obtained for the average needle penetration depth for a sample of bitumen modified with crumb rubber meet the requirements established for bitumen modified with rubber: 45–80 mm^−1^. The softening temperature determines the properties of bitumen at high operating temperatures (the method indirectly characterizes the resistance to rutting) [35]. The results obtained meet the requirements: ≥55 °C. Unmodified bitumen has very low elastic properties (elastic or reversible deformation) at medium and high operating temperatures. An increase in elasticity increases stiffness and hardness. At low temperatures, stiffness decreases and resistance to cracking increases. The results obtained show that the addition of crumb rubber to bitumen significantly increases its elasticity, achieving an elastic recovery of >70%, which corresponds to the >50% figure as regulated in the 2019 Road Standards. The softening point shows the upper limit of the viscosity of bitumen (the ability of bitumen to resist rutting), and the Fraas brittleness temperature shows the lower limit of viscoelasticity (the ability of bitumen to deform at low temperatures and then return to its original state). Elasticity is an instantaneous reversible deformation, while viscoelastic deformation is also reversible; only the return occurs with a delay (delay time is an important parameter of viscosity—the duration of the delay before the material returns to its original shape). The obtained results show that adding crumb rubber to bitumen does not worsen and, in some cases, even improves the brittleness temperature on Fraas by 1–2 °C, which corresponds to a target class of ≤−15 °C.

The viscoelastic properties of rubber-modified bitumen systems (characterized by complex modulus, storage modulus, loss modulus, viscosity, and phase shift angle) were tested over a temperature range of +46 to +94 °C under different shear conditions. The properties of the developed rubber-modified bitumen formulations were compared with the properties of the original B70/100 bitumen, as well as with the properties of SBS-modified bitumen. The obtained data make it possible to judge the rigidity of bitumen systems, as well as the resistance to cracking. In turn, the test procedure, according to AASHTO TP 70 and ASTM D7405, involves testing bitumen systems at a fixed temperature, subjecting them to cyclic loading and unloading. Based on the obtained data, one can judge the elastic properties of bitumen systems (elastic recovery), as well as thermal resistance (including resistance to rutting).

**Figure 2:**
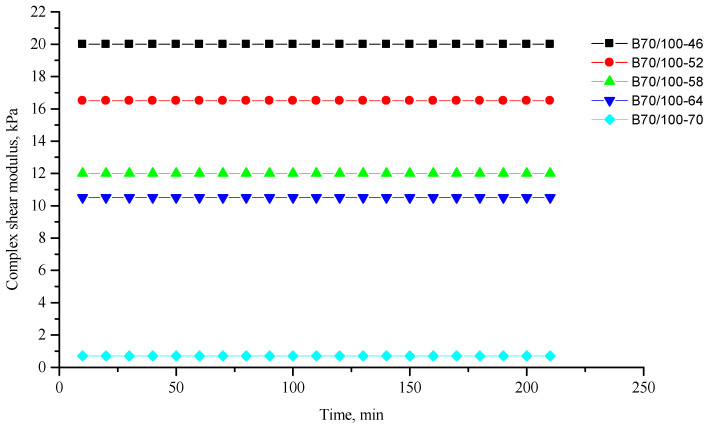
Complex shear modulus G* of the reference unmodified bitumen B70/100 depending on temperature (from +46 to +70 °C) and time.

**Figure 3:**
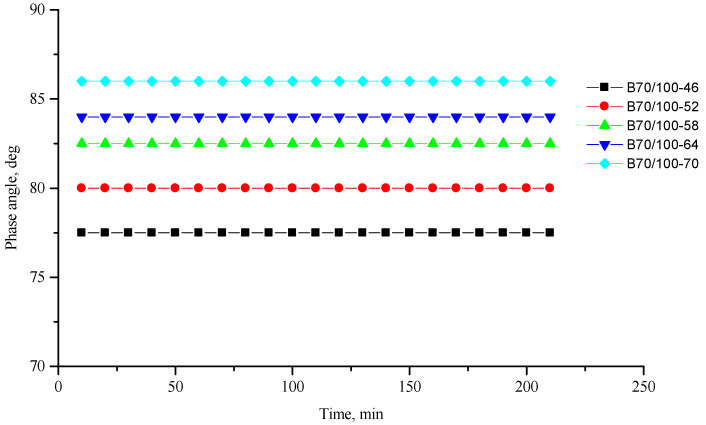
Phase shift angle δ of the reference unmodified bitumen B70/100 depending on temperature (from +46 to +70 °C) and time.

**Figure 4:**
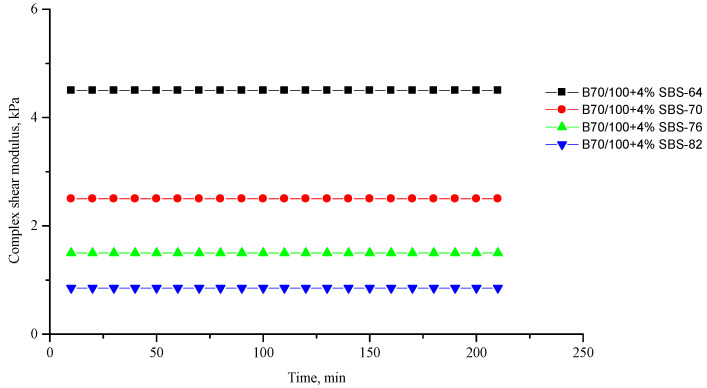
Complex shear modulus G* of the reference SBS-modified (4%) B70/100 bitumen depending on temperature (from +64 to +82 °C) and time.

**Figure 5:**
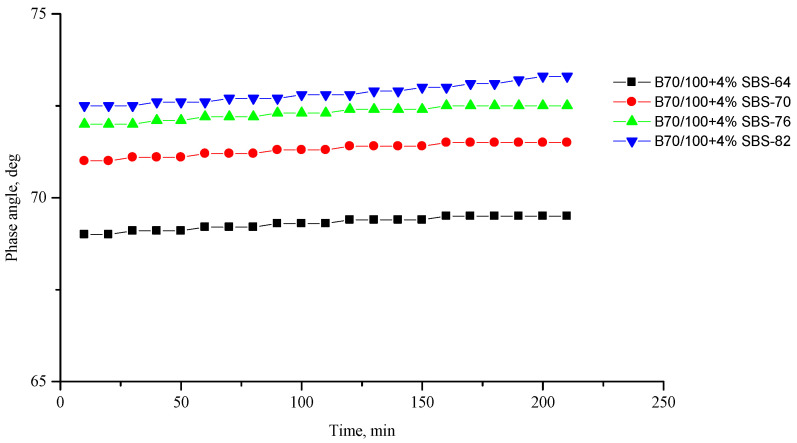
Phase shift angle δ of the reference SBS-modified (4%) B70/100 bitumen depending on temperature (from +64 to +82 °C) and time.

**Figure 6:**
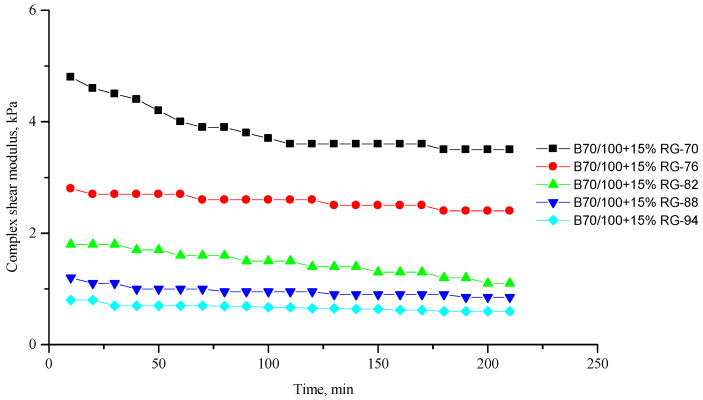
Complex shear modulus G* of the B70/100 bitumen modified with RG crumb rubber (15%), depending on temperature (from +70 to +94 °C) and time.

**Figure 7:**
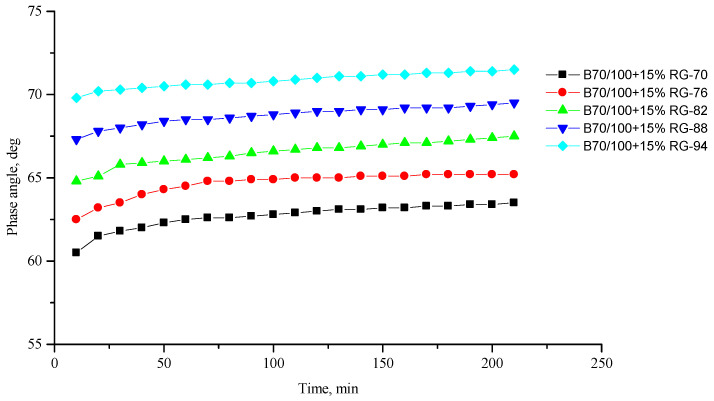
Phase shift angle δ of the B70/100 bitumen modified with RG crumb rubber (15%), depending on temperature (from +70 to +94 °C) and time.

**Figure 8:**
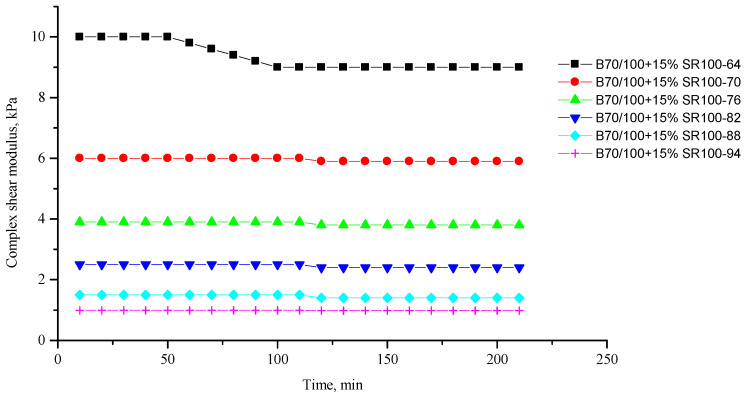
Complex shear modulus G* of the B70/100 bitumen modified with SR100 crumb rubber (15%), depending on temperature (from +64 to +94 °C) and time.

**Figure 9:**
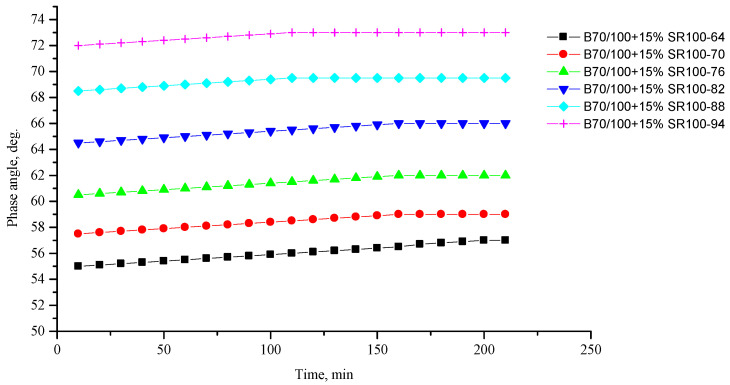
Phase shift angle δ of the B70/100 bitumen modified with SR100 crumb rubber (15%), depending on temperature (from +64 to +94 °C) and time.

**Figure 10:**
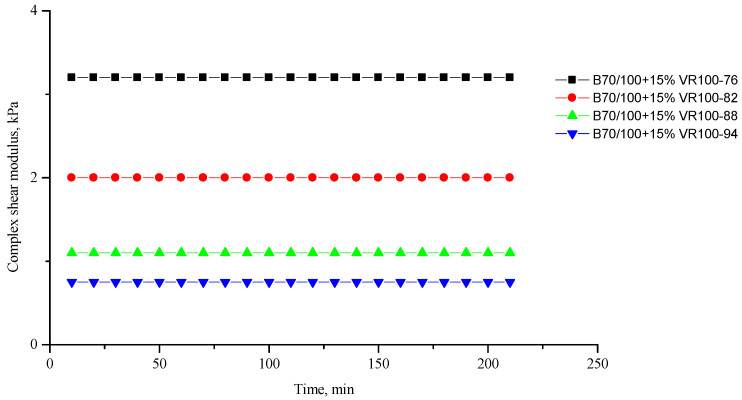
Complex shear modulus G* of the B70/100 bitumen modified with VR100 crumb rubber (15%), depending on temperature (from +76 to +94 °C) and time.

**Figure 11:**
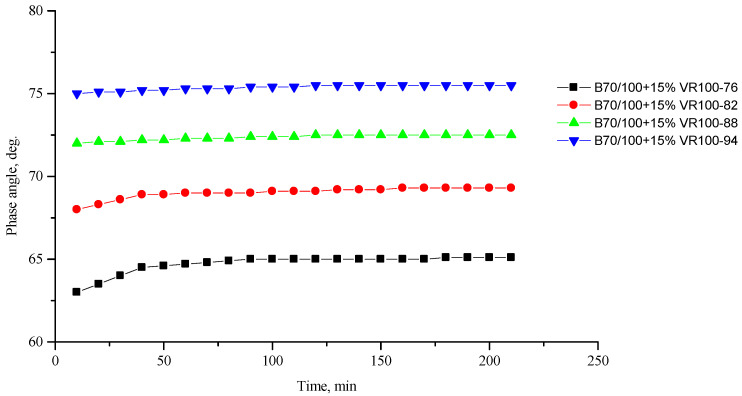
Phase shift angle δ of the B70/100 bitumen modified with VR100 crumb rubber (15%), depending on temperature (from +76 to +94 °C) and time.

**Figure 12:**
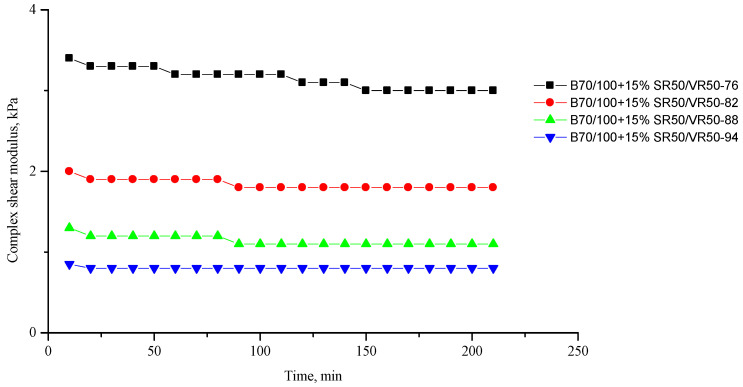
Complex shear modulus G* of the B70/100 bitumen modified with SR50/VR50 crumb rubber (15%), depending on temperature (from +76 to +94 °C) and time.

**Figure 13:**
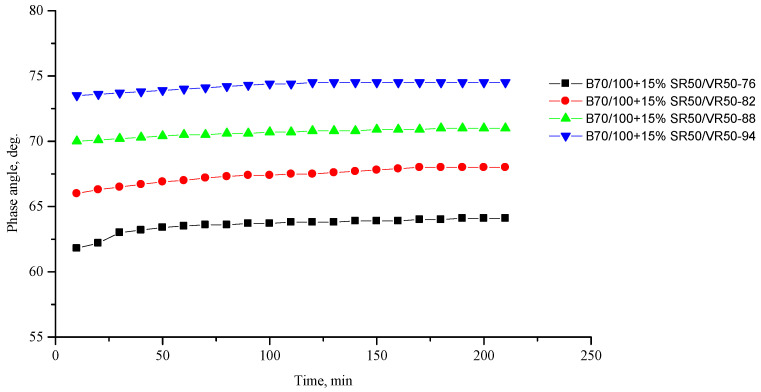
Phase shift angle δ of the B70/100 bitumen modified with SR50/VR50 crumb rubber (15%), depending on temperature (from +76 to +94 °C) and time.

Figure 2, Figure 3, Figure 4, Figure 5, Figure 6, Figure 7, Figure 8, Figure 9, Figure 10, Figure 11, Figure 12 and Figure 13 show the indicators of the complex shear modulus G* and phase shear angle δ of bitumen binders (unmodified B70/100, as well as the bitumen formulations modified with crumb rubber and SBS), depending on temperature and time. With increasing temperature, the phase shift angle δ also increases (elasticity decreases), but the complex shear modulus decreases.

The results of the viscoelastic properties tests performed at this stage show that with increasing temperature, the values of viscosity, shear stress and complex shear modulus of all the studied bitumen systems decrease, and the values of the phase shift angle increase, which is associated with an increase in thermal movement in the system. Therefore, with increasing temperature, the rigidity of bitumen systems decreases and sensitivity to external loads increases, which affects the formation of cracks.

In [35], the difference between filled composites and the original bitumen was shown they are characterized by a monotonous decrease in viscoelastic properties with increasing temperature, while pure bitumen exhibits an inflection point at a temperature of about 30 °C; it has also been shown that at elevated temperatures, the filled composites better retain the stiffness induced by modifications.

When comparing the indicators of the viscoelastic properties of the output bitumen at the same temperature, it is clear that, when SBS is introduced, the rigidity of the system increases significantly (this is indicated by an increase in the complex shear modulus) and, accordingly, the fluidity of the bitumen decreases (this is indicated by a decrease in the phase shift angle—the system stays “harder”).

At the same time, it was observed that the introduction of rubber particles further increases the rigidity of the system and reduces the fluidity of the bitumen system. The greatest influence on the rigidity of bitumen systems is exerted by the introduction of crumb rubber obtained from truck tires (SR100) into the bitumen, while the introduction of particles of fine rubber fraction (RG) has a relatively lesser effect. The obtained results show that it is not the chemical composition of the rubber (passenger car tires (VR100), truck tires (SR100), or a mixture of both (SR50/VR50)) that has a greater influence on the properties of bitumen systems, but rather the size of the rubber particles. It should be noted that introducing all kinds of rubber particles reduce the fluidity of the bitumen system—therefore, one can indirectly judge the decrease in the ease of processing.

It was shown in [35] that thermal stability can be assessed from the temperature dependence of the dynamic modulus using the methodology proposed in the Superpave standard (United States). According to this standard, the maximum operating temperature of a binder is defined as the temperature at which the G*/sinδ value of the material at an angular strain frequency of 10 s^−1^ is at least 1 kPa.

**Figure 14:**
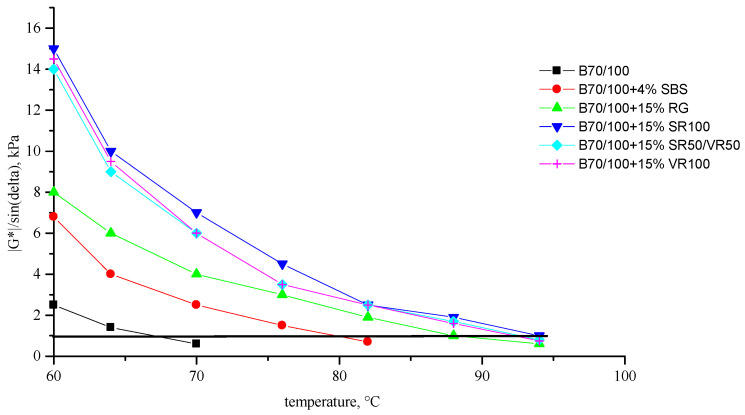
Comparison of the critical temperatures (at |G*|/sinδ = 1 kPa) of the selected rubber-modified bitumen compositions.

Figure 14 shows the values of the highest critical temperatures of the original B70/100 bitumen, the bitumen modified with SBS, and the bitumen formulations modified with various types of waste tire crumb rubber, which indicate rutting resistance (a higher critical temperature indicates greater rutting resistance).

The authors of [35] also showed that introducing SBS or devulcanized crumb rubber significantly increases heat resistance. Moreover, the heat resistance of composites containing crumb rubber and SBS is significantly higher than that of commercial grades of bitumen. It can be concluded that the use of rubber obtained from waste truck tires to modify bitumen allows us to ensure the maximum value of the critical temperature (+94 °C), which is 14 °C higher than that of SBS-modified bitumen (+80 °C), and even 28 °C higher than the output bitumen (+66 °C). It should be noted that the value of the critical temperature of bitumen modified with smaller particles of the rubber fraction is comparatively lower (−88 °C), but it is still 22 °C higher than the indicator characterizing the original bitumen, and 8 °C higher than the composition of the modified bitumen with SBS. Higher critical temperature values of the coarse waste tire rubber particles modified bitumen formulations may be explained by its higher influence on the viscosity and shear modules of the system. This makes the modified bitumen stiffer and thus more resistant to rut-forming.

**Figure 15:**
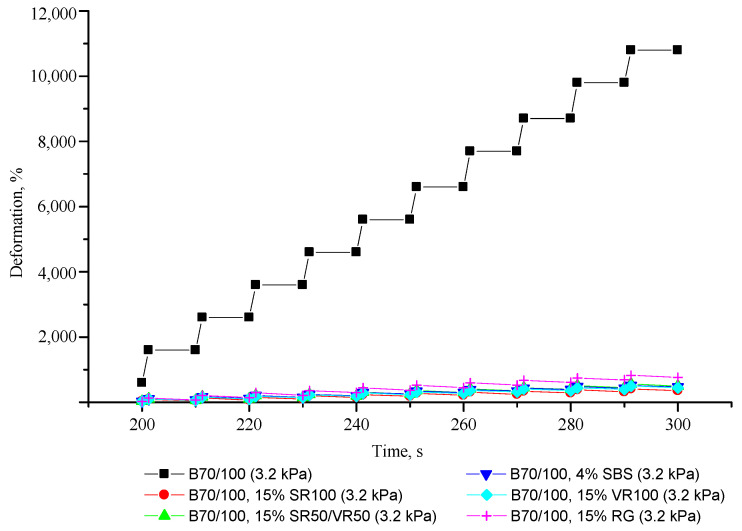
Deformation–time relationship in multiple stress creep-recovery tests.

**Figure 16:**
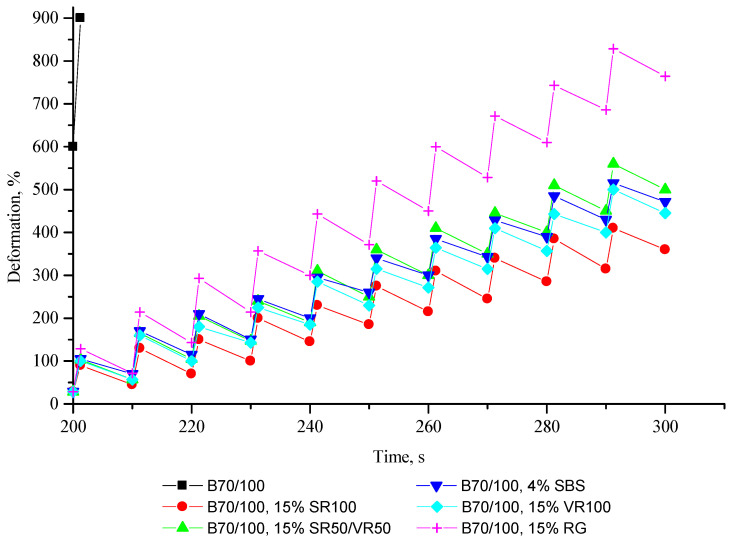
Close-up view of the deformation–time relationships demonstrated in Figure 15.

The resistance of bituminous systems to repeated loading–unloading cycles (MSCR test), which makes it possible to characterize their viscoelastic properties, is presented in Figure 15 and Figure 16.

From Figure 15 and Figure 16, it is clear that unmodified bitumen has no elastic properties, i.e., after loading, the bitumen retains its adopted shape without restoring its original state, which is not optimal from the point of view of reducing the formation of cracks. On the other hand, after adding SBS, as well as all the rubber additives obtained from ELTs, the ability of bitumen to elastic regeneration is significantly improved, and, as a result, the resistance of bitumen to cracking is improved. It can be seen that the effectiveness of the rubber additives used is comparable to the effectiveness of SBS in improving the elastic recovery properties of bitumen. Adding truck tire-derived rubber to bitumen can provide even better elastic recovery properties than adding SBS, while the effectiveness of adding a finer rubber fraction is lower. The stronger influence of adding the more coarse rubber particles to the 70/100-grade bitumen may be explained by the larger particles demonstrating better impact absorption characteristics compared to the finely ground rubber powder. Overall, the results show that a rubber-modifying additive’s effectiveness is mainly affected by particle size, while the chemical composition of crumb rubber has a lesser influence.

## 4. Conclusions

As part of the objective of this research work, the viscoelastic and rheological properties of rubber–bitumen binders obtained using unvulcanized crumb rubber of different sizes and different origins were studied.

The results of the studies showed that with increasing temperature, the values of the complex shear modulus of all studied bitumen binders decrease, and the values of the phase shift angle increase. As a result, with increasing temperature, the rigidity of bitumen systems decreases, and sensitivity to external loads increases; this affects the formation of cracks.

It was also found that when SBS and crumb rubber are introduced into the system, the stiffness of bitumen binders increases significantly, as indicated by an increase in the complex shear modulus. At the same time, introducing rubber particles increase the rigidity of the system more and reduces the fluidity of the bitumen system than when using SBS. The results also show that adding rubber granules to bitumen significantly increases its elasticity, achieving an elastic recovery of >70%. When studying the influence of the size and origin of crumb rubber on the rheological properties of bitumen binders, it was found that it is not the chemical composition of the rubber that has a greater influence on the properties of bitumen systems (passenger car tires, truck tires or a mixture of both), and the size of the rubber particles. Larger particles (2.0–2.5 mm) provide better elastic recovery properties, and, as a result, the bitumen’s resistance to cracking improves. The use of rubber for modifying bitumen, obtained using larger crumb rubbers, regardless of origin, allows for a maximum critical temperature of up to 94 °C, which characterizes the high heat resistance of rubber–bitumen binders and greater rutting resistance.

Thus, the results of the work substantiated the effectiveness of using a larger crumb rubber to modify bitumen for a profitable economic solution since its production costs EUD 150/ton, while the production of traditional rubber powder costs up to EUD 600–800/ton.

## Figures and Tables

**Table 1:** Characteristics of petroleum road viscous bitumen grade B 70/100.

Bitumen Properties	Normative Indicators of the Road Bitumen B 70/100	Actual Value	Test Method
Penetration at 25 °C, 0.1 mm, not lower	87 ± 5	87.2	EN 1426:2015 [54]
Softening point °C, not below	45.8 ± 1.6	45.85	EN 1427:2015 [55]
Brittleness temperature on Fraas °C, not higher	−21 ± 3	−21	EN 12593:2015 [56]
Solubility %, not less	99.75 ± 0.1	99.75	EN 12592:2015 [57]
Flashpoint °C, not below	334 ± 4	335	EN ISO 2592:2006 [58]
Rolling Thin Film Oven Test (RTFOT)			
Change of Mass, %	−0.021 ± 0.01	−0.0212	EN 12607-1:2015 [59]
Increasing in Softening Point °C	7.0 ± 3.6	7.0	EN 1427:2015 [55]
Decreasing in Softening Point °C	52.8 ± 1.6	53.25	EN 1427:2015 [55]
Retained Penetration, %	52 ± 3	51.9	EN 1426:2015 [54]

**Table 2:** Properties of SBS polymer.

Polymer Properties	SBS-01-10
Structure	Linear
Molecular weight, g/mol	150,000
Bound styrene, %	30
Shore A hardness	80
Volatile matter, %	0.8
Ash content, %	0.3
Specific gravity	0.95
Tensile strength, MPa	21
Melt flow index, 200 °C/5 kgf	1

**Table 3:** Bitumen test results.

Bitumen	Penetration at 25 °C, mm^−1^	Brittleness Temperature on Fraas, °C	Softening Point, °C	Elasticity, %	DSR Test, G*/sinδ at 60 °C, kPa	DSR Test, T_crit._ at G*/sinδ = 1 kPa, °C
B70/100	87.0	−21	45.8	-	2.4	66
B70/100 + SBS 2.5%	62.0	−20	60.0	60	-	-
B70/100 + SBS 4%	50.4	−17	66.3	88	6.8	80
B70/100 + 15% RC	54.6	−22	66.8	>70	8	89
B70/100 + 15% SR100	49.4	−23	70.0	>70	14.4	94
B70/100 + 15% SR50/VR50	47.6	−22	69.2	>70	12	91
B70/100 + 15% VR100	52.3	−21	65.9	>70	13.2	90
Target value	45–80	≤−15	≥55	≥50	≥1	-

## Data Availability

Data are contained within the article.
